# A novel prognostic index based on the analysis of glycolysis-related genes in idiopathic pulmonary fibrosis

**DOI:** 10.1097/MD.0000000000033330

**Published:** 2023-03-17

**Authors:** Yu Li, Yaju Deng, Jie He

**Affiliations:** a Department of Pulmonary and Critical Care Medicine, The Second Affiliated Hospital of Guangxi Medical University, Nanning, Guangxi, PR China; b Emergency Department, Guangxi District Maternal and Child Health Hospital, Nanning, Guangxi, PR China; c Clinical Medical College of Chengdu Medical College, Chengdu, Sichuan, PR China; d Department of Pulmonary and Critical Care Medicine, The First Affiliated Hospital of Chengdu Medical College, Chengdu, Sichuan, PR China.

**Keywords:** genes, glycolysis, idiopathic pulmonary fibrosis, interstitial lung disease, prognostic

## Abstract

Idiopathic pulmonary fibrosis (IPF) is a lung disease that is both chronic and progressive and is characterized by glycolysis. However, glycolysis’s function and its clinical significance in IPF are still not well understood.

We accessed the Gene Expression Omnibus database to retrieve mRNA expression information for lung tissue and other samples. We identified genes associated with glycolysis that had differential expression levels between IPF and controls. In this work, we conducted a comprehensive bioinformatic analysis to systematically examine the glycolysis-associated genes with differential expression and subsequently investigated the possible prognostic significance of these genes. Additionally, the expression profiles of the associated prognostic genes were further investigated via quantitative real-time polymerase chain reaction in our cohort. In this investigation, we found that the expression of 16 genes involved in glycolysis was differentially expressed. Among them, 12 were upregulated and 4 were downregulated. We found that 3 glycolysis-related genes (stanniocalcin 2, transketolase like 1, artemin) might serve as hub genes for anticipating patient prognosis. The data from these genes were used to generate the prognostic models. The findings confirmed that high-risk IPF patients recorded a shorter overall survival relative to low-risk patients. This prognostic model yielded 1-, 2-, and 3-year survival rates of 0.666, 0.651, and 0.717, correspondingly, based on the area under the curve of the survival-dependent receiver operating characteristic. The GSE27957 and GSE70866 cohorts validated these findings, indicating the model has a good predictive performance. All 3 glycolysis-associated genes were validated to be expressed in our cohort. Finally, we used mRNA levels from 3 genes to produce a nomogram to quantitatively predict the prognosis of IPF individuals. As possible indicators for the prognosis of IPF, the glycolysis-related genes stanniocalcin 2, transketolase like 1, and artemin were shown to be promising candidate markers.

## 1. Introduction

Idiopathic pulmonary fibrosis (IPF) has been recognized as a chronic, irreversible, progressive interstitial lung disorder with undetermined etiology and poor clinical prognosis.^[1]^ The incidence of IPF is low in young people and more common in the elderly. The median survival duration following diagnosis ranges between 2 and 4 years.^[2,3]^ Due to the development and use of antifibrotic drugs (pirfenidone or nintedanib) in recent years, the prognosis of IPF individuals has improved to some degree.^[4]^ However, IPF is still incurable, in part because its etiology is complex and the mechanism is unclear.^[5]^ Therefore, further comprehension of the molecular basis that underlies IPF is crucial for improving the prognosis, early screening, and diagnosis of IPF patients.

Pulmonary fibrosis is also a metabolic-related disease, and the changes in glucose metabolism perform an integral function in the formation of pulmonary fibrosis.^[6,7]^ Fibrosis may develop as a consequence of lung macrophage activation and subsequent inflammation, which might in turn induce a proteolytic phenotype in macrophages and reduce lung elastin levels. Active macrophages rely heavily on glycolysis and high lactate generation to swiftly supply the energy needed to sustain the inflammatory response.^[8]^ Aerobic glycolysis is commonly referred to as the Warburg effect where cells can still carry out glycolysis under the condition of sufficient oxygen, convert glucose into pyruvate, and process it into lactic acid, consequently promoting cell growth and proliferation.^[9]^ Researchers have indicated that in pulmonary fibrosis, aerobic glycolysis is considerably enhanced. It has been shown that inhibiting aerobic glycolysis may alleviate bleomycin-mediated lung fibrosis and reduce the rate of TGF-β-induced collagen formation.^[10,11]^ The results of Xie et al’s study also showed that lung fibroblasts had enhanced glycolysis and upregulation of glycolysis enzymes in myofibroblasts that have been differentiated in vitro by TGF-β.^[11]^ The findings of Kottmann’s research indicated the presence of lactic acid accumulation in patients with IPF.^[10]^ The above research data suggest that the process of aerobic glycolysis of lung tissue might cause fibrosis by activating TGF- β and other fibrogenic factors through the survival and accumulation of lactic acid. Up to now, the pathogenicity of glycolysis in IPF is still worthy of further study. A better comprehension of the role glycolysis performs in the IPF has led to the accumulation of more data supporting the treatment of patients with the disease. The results of the investigation by Yin et al^[12]^ indicated that when bleomycin-mediated pulmonary fibrosis mice were treated with HK2 inhibitors, the expression of fibrogenic genes was reduced and lung function remained stable. Pulmonary fibrosis is associated with glycolysis, however, the specific molecular mechanisms that underly glycolysis in the lungs are not well characterized. Therefore, the study on the systematic function of glycolysis-associated genes in IPF is warranted to elucidate the function of glucose metabolism in IPF and find novel targets for IPF treatment.

This research aimed to examine the functions of glycolysis in IPF and assess the prospective clinical value of differentially expressed glycolysis-associated genes for prognostic stratifications. In particular, the Gene Expression Omnibus (GEO) database was searched for data on mRNA expression and clinicopathological characteristics. We conducted bioinformatics analysis to identify genes involved in glycolysis with differential expression between individuals with IPF and healthy controls and extensively analyzed the possible biochemical processes and functions of these genes. Lastly, we combined glycolysis-related gene expression data with clinical data to examine the possible influence of these genes on prognosis.

## 2. Method

### 2.1. Data collection and analysis

The molecular signature dataset (MSigDB, http://www.broad.mit.edu/gsea/msigdb/) is a repository of annotated gene sets for Gene Set Enrichment Analysis. Four glycolysis-associated gene sets were extracted, containing KEGG GLYCOLYSIS GLUCONEOGENESIS, BIOCARTA GLYCOLYSIS PATHWAY, HALLMARK GLYCOLYSIS, and REACTOME GLYCOLYSIS^[13]^ (Supplementary Table S1, Supplemental Digital Content, http://links.lww.com/MD/I688). An aggregate of 255 glycolysis-associated genes was retrieved from MSigDB, which offers gene annotations in great depth. We retrieved the Next Generation Sequencing data of 103 IPF and 103 control lung tissues with corresponding clinical data from GSE 150910.^[14]^ Information on patients in the GSE150910 dataset is displayed in Table 1. This dataset was used for further analysis and mining. The limma R package (https://bioconductor.org/packages/limma/) was utilized to execute the negative binomial distribution technique with the absolute value of log_2_(fold change) > 1.0 and false discovery rate < 0.05 to find differentially expressed glycolysis-associated genes between IPF and control lung tissues. The most common recursive feature-removal algorithm, support vector machine-recursive feature elimination (SVM-RFE),^[15]^ was utilized to choose the features for removal. The differentially expressed genes were used to complete SVM-RFE analysis, which could be used to screen the optimal feature genes in the GSE150910 dataset.

**Table 1:** Clinical characteristics of all participants in GSE150910.

Characteristics	IPF (N = 103)	Control (N = 103)	*P* value
Age, yr	60.3 ± 8.3	59.9 ± 10.2	.758
Sex	N = 103	N = 103	.941
Male	57 (55%)	45 (44%)	
Female	46 (45%)	58 (56%)	
Race	N = 101	N = 103	.061
Non-Hispanic White	85 (84%)	87 (84%)	
Hispanic	7 (7%)	4 (4%)	
Asian	2 (2%)	3 (3%)	
Black	4 (4%)	9 (9%)	
Other	3 (3%)	0 (0%)	
Smoke	N = 95	N = 96	.71
Ever	40 (42%)	43 (45%)	
Never	55 (58%)	53 (55%)	
Sampling method			.471
Surgical lung biopsy	36 (35%)	41 (40%)	
Transplant	67 (65%)	62 (60%)	

IPF = idiopathic pulmonary fibrosis.

### 2.2. Protein-protein interaction (PPI) network

The glycolysis-associated genes showing differential expression were imported into the Search Tool for the Retrieval of Interacting Genes (https://string-db.org/cgi/input.pl) for anticipating the PPI network. In the meantime, the R software (version 3.6.1; R Foundation for Statistical Computing, Vienna, Austria) was employed to visualize the PPI network.

### 2.3. Glycolysis-related prognostic model construction

The GSE28042 provided us with microarray expression matrix data for 75 IPF patients with matching clinical information.^[16]^ The information on the genechip is displayed in Table 2. We subsampled 100% of the GSE28042 dataset for model building. Premised on the survival R package, we used univariate cox regression analysis and the log-rank test to identify the important risk variables in all optimum feature genes. To eliminate the possibility of missing important information, we adjusted the cutoff *P* value to 0.2 and selected 3 genes associated with survival as candidates for further investigation. Following that, to evaluate the predictive outcomes of IPF patients and to calculate risk scores, we developed a multivariate cox regression model. The following is an equation used to calculate each sample’s risk score based on the aforementioned potential candidate optimal feature genes:

**Table 2:** Clinical information of IPF patients included in GSE28042, GSE27957, and GSE70866.

GSE number	Samples	IPF age (yr)	Country	Ethnicity	Platform	Authors
GSE28042	75 IPF samples	69.00 ± 8.16	USA	Caucasian	GPL6480	Herazo et al
GSE27957	45 IPF samples	67.10 ± 8.20	USA	Caucasian	GPL5175	Huang et al
GSE70866	176 IPF samples	62.00 ± 6.00	Germany	Caucasian	GPL14550/GPL17077	Prasse et al

IPF = idiopathic pulmonary fibrosis.

Risk score = β1*Exp1 + β2* Exp2+β3* Exp3+…,

where β signifies the coefficient value and Exp indicates the levels of gene expression. Subsequently, the median approximated risk scores were applied to classify IPF patients into low- and high-risk groups (categories). The “prcomp” function included in the “stats” R package was applied to conduct the principal component analysis of the 3-gene signature. Next, the log-rank test was conducted to contrast the prognostic results of the 2 cohorts. We investigated the predictive power of the aforementioned model via receiver operating characteristic (ROC) analysis utilizing the SurvivalROC R package (https://CRAN.R-project.org/package=survivalROC).

### 2.4. Verification of the prognostic model

The GSE27957(45 IPF patient samples with matching prognosis)^[17]^ and GSE70866(176 IPF patient samples with matching prognosis)^[18]^ datasets were employed to verify the estimated findings and assess the model’s prognostic performance. The information on the genechip is shown in Table 2. R-Rroject (R Foundation for Statistical Computing, Vienna, Austria) was used to evaluate the genechip data, and the statistics of chip signal intensity distribution and relative logarithmic signal intensity were applied to assess the quality of genechip data.

### 2.5. Ethics

Ethical approval was obtained from the ethics committee of the First Affiliated Hospital of Chengdu Medical College (IRB ID:2021CYFYIRB-BA-32–01). Each subject signed the informed consent.

### 2.6. Validation of 3 significant differentially expressed glycolysis-associated genes via quantitative real-time polymerase chain reaction (qRT-PCR)

We likewise utilized real-time PCR to verify the expression of the 3 prognostic genes in the research cohort. Beginning July 2021 to September 2022, we acquired 15 peripheral blood mononuclear cells (PBMCs) from IPF patients and 15 PBMCs from healthy individuals at the First Affiliated Hospital of Chengdu Medical College in Sichuan, China. The eligible subjects for this research included adult patients clinically diagnosed with IPF as per the European Respiratory Society guidelines/American Thoracic Society/(2011).^[19]^ The TRIzol reagent (Invitrogen, Carlsbad, CA) was used for the extraction of total RNA from PBMCs. After the determination of total RNA purity and concentration, reverse transcription of the total RNA into cDNA was done utilizing the PrimeScript RT reagent kit (Takara, Japan). SYBR Green Premix Ex Taq II was utilized to execute the qRT-PCR. The PCR parameters were as illustrated below: 95°C for 30 seconds, ensued by 40 cycles of 95°C for 5 seconds, and 60°C for 30 seconds for each particular primer. Lastly, the 2^−△△Ct^ method was applied to derive the relative mRNA expression profiles of the 3 genes. Table 3 lists the primer sequences. The median with interquartile range was used to present the findings, and nonparametric tests (Kruskal–Wallis or Mann–Whitney tests) were employed for comparisons among cohorts. *P* values < .05 denoted the significance criterion.

**Table 3:** Specific primer sequences used in quantitative real-time polymerase chain reaction.

Gene	Primer sequences (5′-3′)
STC2	F: CAGAATACAGCGGAGATCCAGCAC
	R: GTCAGCAGCAAGTTCACGAGGTC
TKTL1	F: AGGATGGCTCGGACAAGGACTG
	R: ACCACATAAGTGTTCCACCCAAAGG
ARTN	F: TGCACCCCATCTGCTCTTC
	R: ACGGTTCTCCAGGTGCTGTT
GAPDH	F: CAGCCGCATCTTCTTGTGC
	R: GGTAACCAGGCGTCCGATA

ARTN = artemin, GAPDH = glyceraldehyde-3-phosphate dehydrogenase, STC2 = stanniocalcin 2, TKTL1 = transketolase like 1.

### 2.7. Construction of the nomogram

To elucidate a quantitative method to predict an IPF patient’s probability of glycolysis-related genes, we constructed a nomogram based on the “rms” R program.

## 3. Results

### 3.1. Determination of glycolysis-associated genes with differential expression

**Figure 1.**
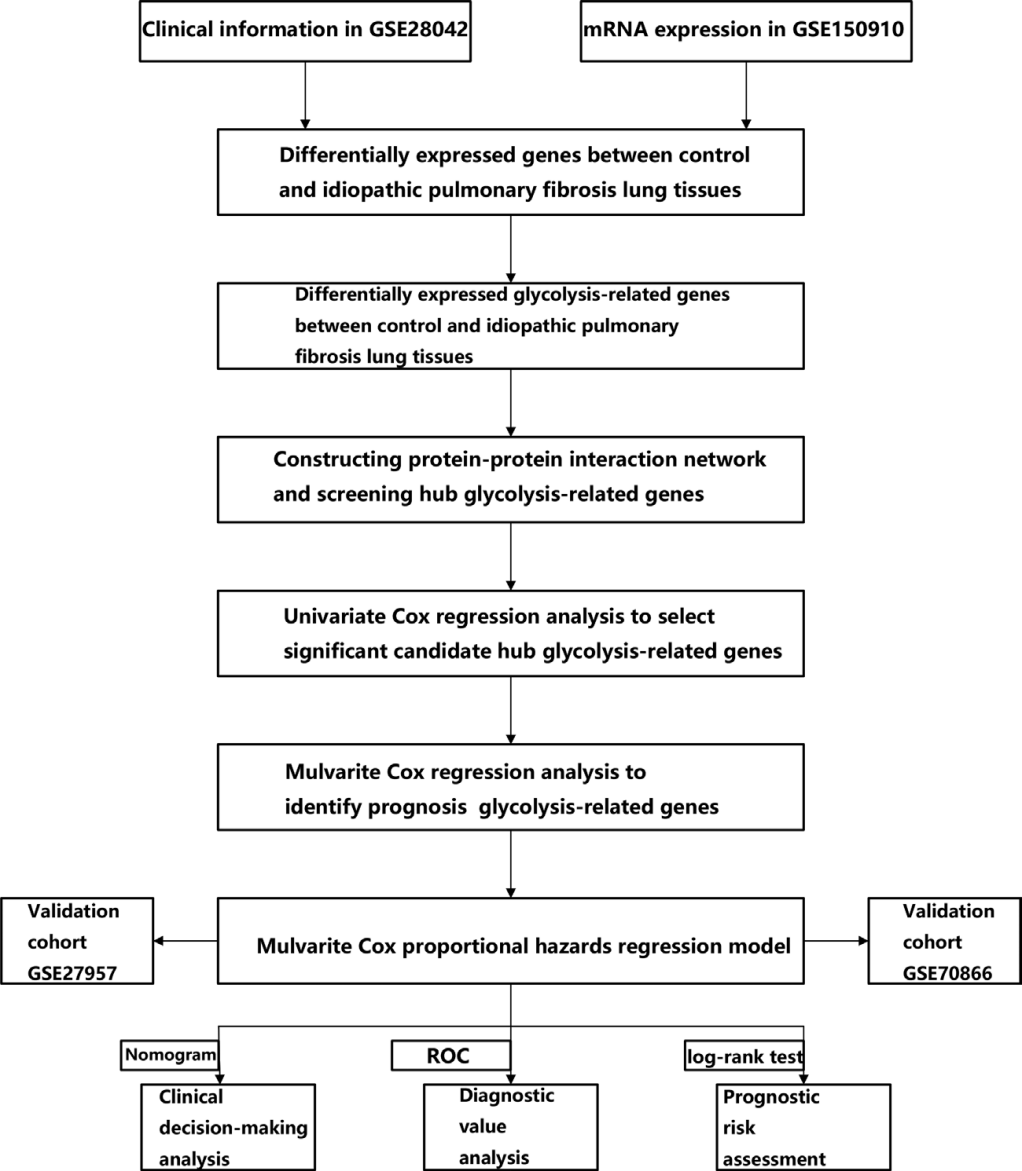
The process flow for evaluating the glycolysis signature in idiopathic pulmonary fibrosis.

**Figure 2.**
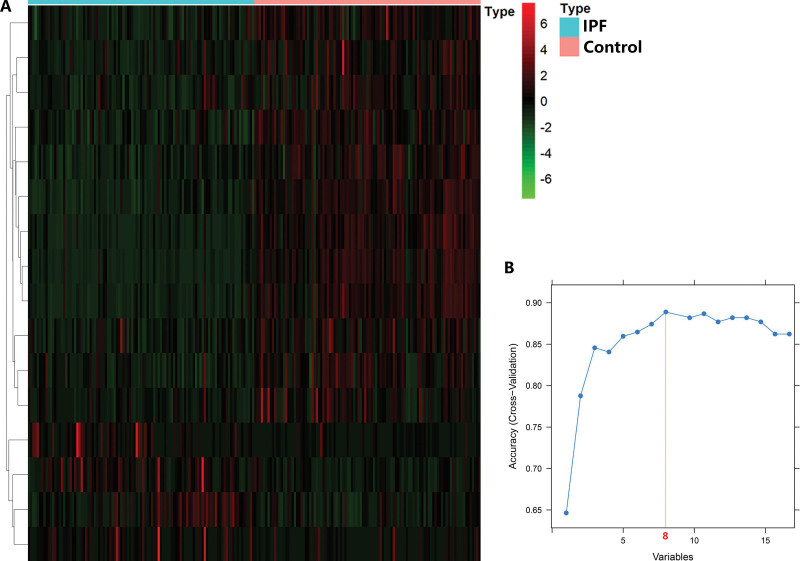
Genes involved in glycolysis showing differential expression in idiopathic pulmonary fibrosis. (A) A heatmap of glycolysis-associated genes with differential expression. (B) Support vector machine-recursive feature elimination for screening the optimal feature glycolysis-related genes.

**Figure 3.**
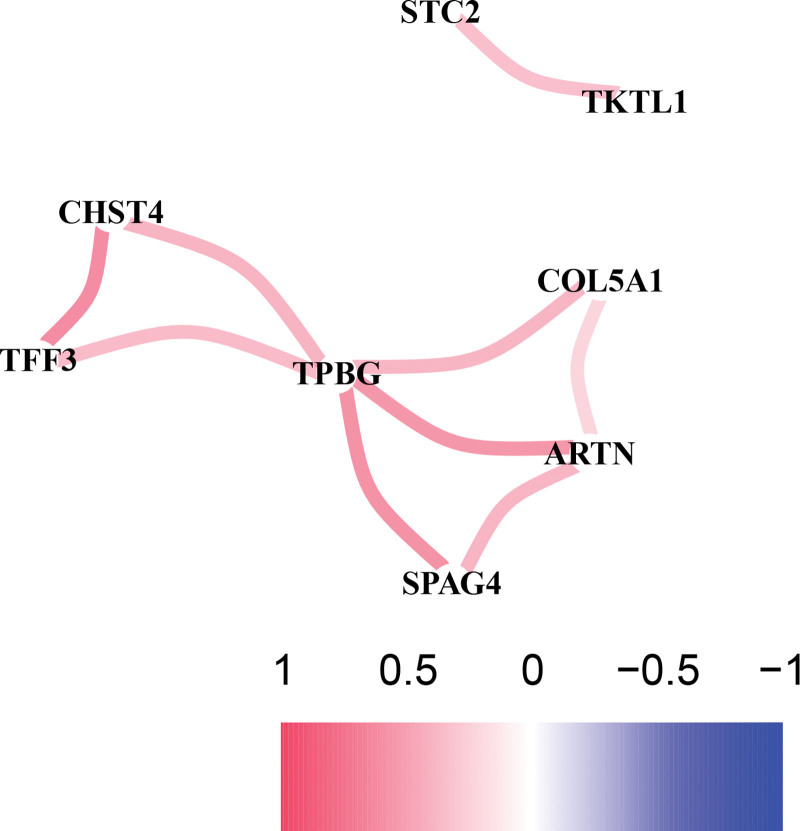
The network of correlations between the genes that are linked to glycolysis (red and blue lines denote positive and negative correlations, correspondingly).

Genes involved in glycolysis were systematically analyzed to determine their potential predictive performance and key roles in IPF. The investigation was carried out exactly as planned, as illustrated in Figure 1. The IPF patient database that was compiled using GEO, comprised 103 IPF and 103 control lung tissues. The R programs were utilized to analyze the data and identify differentially expressed genes implicated in glycolysis. As per the assessment criteria, there were 16 genes involved in glycolysis that showed differential expression between IPF patients and controls, with 12 upregulated and 4 downregulated genes. As illustrated in Figure 2A, the outcomes were depicted in the heatmap. Next, the SVM-RFE method was used to identify 8 genes among those with a differential expression that are involved in glycolysis (Fig. 2B). To further examine the interplay of the identified glycolysis-associated genes, we conducted a PPI analysis. Figure 3 illustrates the correlation network that consists of 8 glycolysis-associated genes that exhibit differential expression levels.

### 3.2. Prognosis-related glycolysis-related genes selecting

**Figure 4.**
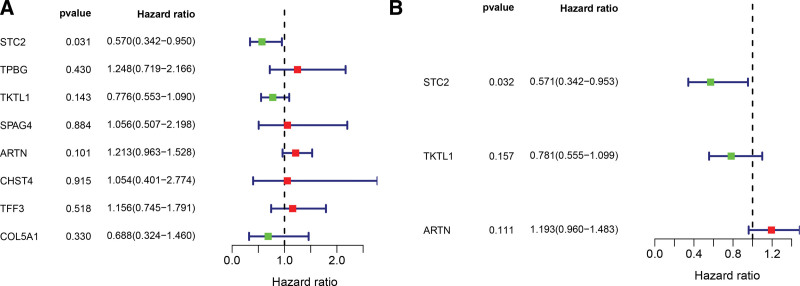
The use of the Cox regression model allowed for the discovery of glycolysis-associated genes with prognostic significance. (A) Univariate Cox regression analysis, (B) Multivariate Cox regression analysis.

To find glycolysis-associated genes that have a significant link to overall survival (OS), a univariate analysis that was based on hub glycolysis-related genes was conducted. As per the hazard ratio forest map, there was a strong link between the expression of 8 glycolysis-associated genes and the IPF patients’ prognoses (Fig. 4A). The 8 glycolysis-related genes were then examined by multiple stepwise Cox regression to evaluate the influence of these genes on survival duration and clinical outcomes. As illustrated in Figure 4B, stanniocalcin 2 (STC2), transketolase like 1 (TKTL1), and artemin (ARTN) were discovered to be independent predictors for IPF patients.

### 3.3. Development of the model and evaluation for prognosis-associated genetic risk score

The glycolysis-related prediction model was built using the 3-hub glycolysis-associated genes discovered in the preceding analysis. Each IPF patient’s risk score was determined as illustrated below:

Risk score = (−0.561*ExpSTC2) + (−0.247*ExpTKTL1) + (0.177*ExpARTN).

**Figure 5.**
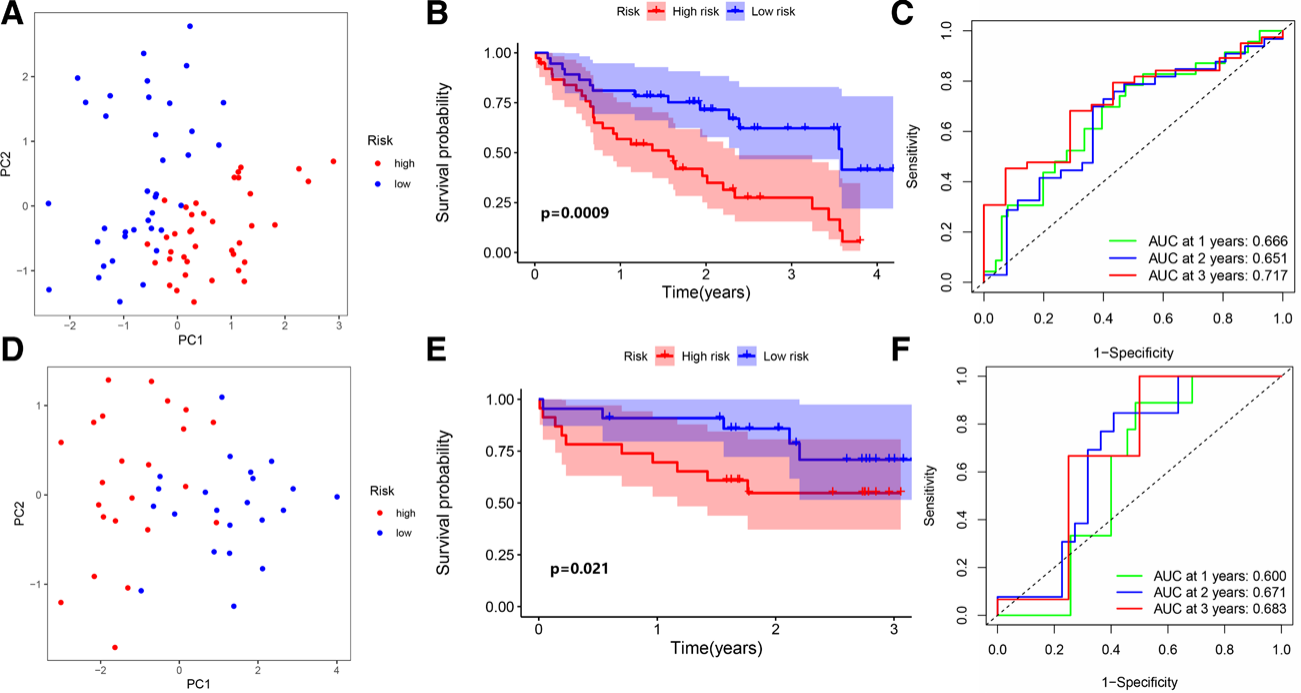
Three prognostic models premised on glycolysis-associated genes were analyzed using risk scores in the GSE28042 and GSE27957 cohorts. (A) Risk score-based PCA for individuals with IPF in the GSE28042 cohort. (B) The GSE28042 cohort-related survival distribution for patients with high- and low-risk scores. (C) ROC curve for forecasting overall survival in the GSE28042 cohort, (E) Patients with IPF in the GSE27957 cohort, shown on a PCA plot as per their risk scores. (F) Survival distribution of GSE27957 patients classified into high- and low-risk groups. (G) Anticipating overall survival in the GSE27957 cohort using the ROC curve. IPF = idiopathic pulmonary fibrosis, PCA = principal component analysis, ROC = receiver operating characteristic.

Subsequently, we performed a survival analysis to evaluate how well this model predicted patient outcomes. By using the median of their risk scores, 75 individuals with IPF were categorized into high- or low-risk categories. The principal component analysis illustrated that patients exposed to varying risks were grouped into 2 cohorts (Fig. 5A). According to the findings, IPF patients exhibiting elevated risk scores recorded a dismal prognosis as opposed to those with lower-risk scores (Fig. 5B). The prognostic performances were evaluated utilizing survival-dependent ROC analysis (Fig. 5C). The area under the ROC curve value for 1-, 2-, and 3-year survival rates were 0.666, 0.651, and 0.717, consecutively, demonstrating a modest prognostic ability of hub glycolysis-related genes in patient survival prediction.

### 3.4. Prognostic model’s validation

**Figure 6.**
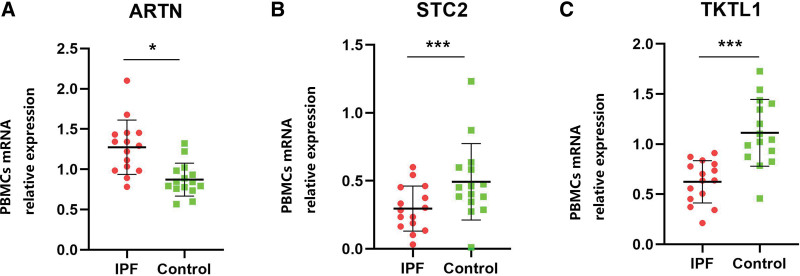
Three prognostic genes with differential expression in IPF patients’ PBMCs. (A) ARTN, (B) STC, and (C) TKTL1. The data are expressed as median with interquartile range (IQR) (Mann–Whitney test). ***P < .001, **P < .001, and *P < .05 represented significant findings. ARTN = artemin, IPF = idiopathic pulmonary fibrosis, PBMCs = peripheral blood mononuclear cells, STC = stanniocalcin, TKTL1 = transketolase like 1.

The GSE27957 as well as the GSE70866 datasets were analyzed to verify the prognostic accuracy of the 3 glycolysis-associated genes prediction model in IPF groups. In the GSE27957 and GSE70866 datasets, we observed that high-risk patients experienced dismal OS in contrast with low-risk patients (Fig. 5D– F; Supplementary Figure S1A–C, Supplemental Digital Content, http://links.lww.com/MD/I689). These findings suggested that this predictive model predicated on glycolysis-related genes exhibited good specificity and sensitivity. Additionally, we validated the expression of the 3 key genes in the study sample. As projected, STC2 and TKTL1 were significantly decreased in IPF patients, while ARTN was increased (Fig. 6A–C).

### 3.5. Three hub glycolysis-related genes were used to generate a nomogram

**Figure 7.**
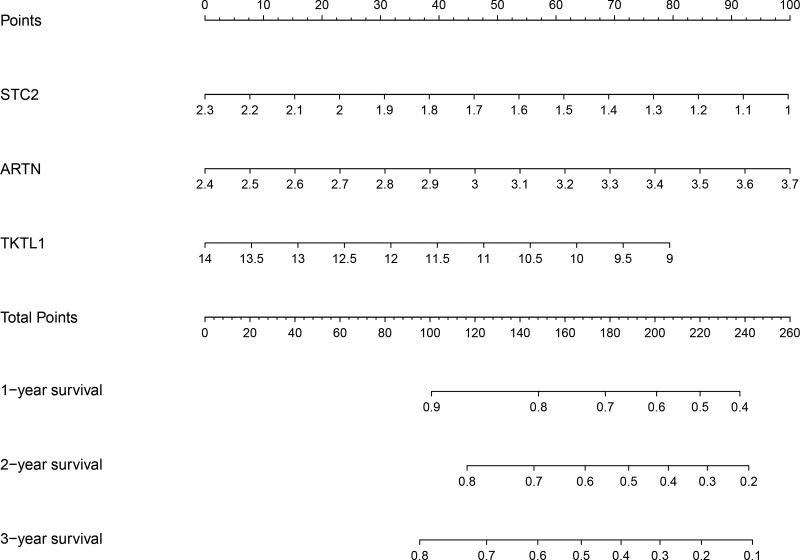
One, 2, and 3-year survival nomograms for patients with IPF in GSE28042. IPF = idiopathic pulmonary fibrosis.

To facilitate the formulation of quantitative techniques for IPF prognosis, we constructed a nomogram centered on hub genes associated with glycolysis (Fig. 7). Relying on the multivariate stepwise Cox regression analysis, points were assigned to each of the variables utilizing the point scale in the nomogram. We computed each IPF patient’s aggregate score by adding the points from 3 variables. Moreover, by comparing the computed points in our nomogram with the survival rate, we may effectively anticipate the 1-, 2-, and 3-year survival chances of IPF patients.

No comprehensive investigation of glycolysis-associated genes has been conducted to evaluate the clinical signature of IPF because of the complexity and controversies regarding the function of glycolysis in IPF progression. In terms of glycolysis, we discovered and confirmed 3 prognostic glycolysis-related genes. The findings suggest that the prognosis of IPF patients may be stratified using a model centered on the expression of 3 genes involved in glycolysis, allowing for more personalized therapy depending on patient risk.

## 4. Discussion

In this research, we found 16 glycolysis-related genes with differential expression between IPF and control lung tissues based on GSE150910 obtained from the GEO database. GSE150910 is the most recent dataset, covering the largest sample size. We also used the SVM-RFE for screening the optimal glycolysis-related genes. Then, we built a co-expression network and PPI network of these genes. This led to the identification of 8 hub genes involved in glycolysis. Many of the hub glycolysis-associated genes were previously linked to the advancement and prognosis of fibrosis.^[20–22]^ Specifically, 3 glycolysis-related genes were discovered as being linked to the prognosis of IPF, including STC2, TKTL1, and ARTN.

The 2 ubiquitous states of cellular energy metabolism are glycolysis and oxidative phosphorylation.^[23]^ Even though glycolysis is not as effective as oxidative phosphorylation in the creation of ATP, it is still capable of oxidizing glucose faster and increasing the generation of lactic acid.^[24]^ At the same time, mounting research data indicates that aerobic glycolysis occurs both in tumors and normal cells, and its effect is beyond energy production. The metabolites of aerobic glycolysis also perform an imperative function in modulating cell function, particularly cellular proliferation, synthesis of the extracellular matrix, autophagy, and apoptosis.^[25]^ Lipopolysaccharide stimulation of alveolar epithelial cells, according to the findings of certain research, may enhance the process of aerobic glycolysis, accelerate the synthesis of the metabolite lactic acid, and promote the release of inflammatory cytokines, all of which contribute significantly to acute lung injury.^[26]^ Other research reports have indicated that through activation of the PI3K-Akt-mTOR/PFKFB3 pathway and aerobic glycolysis, lipopolysaccharide promotes type I collagen production in lung fibroblasts.^[27]^ Therefore, glycolysis serves an instrumental function in IPF occurrence and development. The screened STC2, TKTL1, and ARTN may broaden our understanding of the mechanism of IPF.

STC, which is a hormone containing a glycosylated peptide, initially identified in a bony fish was previously demonstrated as having an integral function in phosphate and calcium homeostasis.^[28]^ Research evidence has indicated that 2 STC paralogs (STC1 and STC2) in mammals exhibit a widespread expression in various tissues.^[29,30]^ On the human chromosome, the STC2 gene is found at position 5q35.1. In both humans and mice, STC2 is made up of 4 exons that span 13kb of DNA.^[31]^ Upregulation of STC2 in N2a mice neuroblastoma cell lines after being subjected to thapsigargin- and tunicamycin-stimulated estrogen receptor stress was the first piece of evidence for STC2’s cytoprotective role, presented by Thinakaran et al.^[28]^ An upregulation of STC2 was also observed following hypoxia and oxidative stress mediated by H2O2. When STC2 was overexpressed, it protected cells against thapsigargin-stimulated cell death in both HeLa and N2a cell lines.^[32]^ Kim et al^[33]^ demonstrated that the overexpression of STC2 triggered the pERK1/2 and pAKT signaling pathways, protecting cells from oxidative stress-triggered cell injury. In our study, bioinformatics analysis and qRT-PCR results suggested that STC2 was lowly expressed in IPF. Cox univariate regression analysis showed that the hazard ratio value of STC2 was 0.570, suggesting that STC2 was a protective factor. We hypothesized that after STC2 expression was reduced, the protective effect on alveolar epithelial cells was lost, and alveolar epithelial cells were over-repaired after repeated injury and converted to fibroblasts, which finally led to the formation of pulmonary fibrosis. It is still worthwhile to investigate STC2’s function in pulmonary fibrosis.

TKTL1 is one of the 3 members of the human TKT gene family (TKT, TKTL1, and TKTL2).^[34]^ In the pentose phosphate pathway, TKTL1 is a rate-limiting enzyme that is abundantly expressed in epithelial-derived normal and tumor cells. Consequently, TKTL1 performs an integral function in the survival, growth, proliferation, migration, and differentiation of cells, and participates in the incidence and advancement of numerous human illnesses.^[35–37]^ At present, extensive research points to a strong association between TKTL1 and malignant growth. Although TKTL1 is overexpressed in non-small cell lung cancer, it is unrelated to patient outcomes in terms of OS, disease-free survival, or tumor node metastasis stages.^[38]^ In this study, TKTL1 is a protective factor in IPF. Verified by qRT-PCR, TKTL1 is lowly expressed in PMBCs of IPF patients. There is currently no investigation conducted on the link between TKTL1 and IPF. Whether TKTL1 participates in the formation and development of IPF is worthy of further study.

ARTN is a well-recognized affiliate of the glial cell line-derived neurotrophic factor ligand family (glial cell line-derived neurotrophic factor-family ligands). Its signal transduction mainly regulates intracellular signal transduction by combining with the co-receptor GFR α 3-RET to regulate intracellular signal transduction, thus affecting neural system development and cell homeostasis.^[39]^ Ceyhan et al^[22]^ showed that in chronic pancreatitis, GFRalpha3 and ARTN exhibited a substantial overexpression and were sited in neural ganglia, Schwann cells, and smooth muscle cells of arteries. Also, the elevated ARTN mRNA levels were found to correlate with hypertrophy, neural density, perineural inflammatory cell infiltration, inflammation, and pain severity. In addition, both ARTN expression and neural alteration exhibited a positive link to the severity of fibrosis. Pancreatic fibrosis and pulmonary fibrosis have some similarities in pathological features and mechanisms, which are caused by repeated damage of epithelial cells, leading to the formation of scars in tissue.^[40]^ The results of bioinformatics analysis in this research illustrated that ARTN was increasingly expressed in IPF tissues, and ARTN had a negative correlation with the prognosis of IPF. Combined with the previous literature, we speculate that ARTN might be implicated in the inflammatory cell infiltration in pulmonary fibrosis and promote the formation of IPF. Song et al^[41]^ illustrated that ARTN may enhance lung cancer cell migration and invasion, which might be related to hypoxia-induced ARTN-promoting epithelial-mesenchymal transition (EMT) through AKT signaling. In addition to being related to tumor invasion and metastasis, EMT is also involved in typical wound healing and performs a function in the excessive repair of tissues in IPF.^[42,43]^ Therefore, we believe that ARTN might be implicated in the EMT process of IPF. This research verified the high expression of ARTN in PBMCs of IPF patients by qRT-PCR, but further research is needed to improve the mechanism.

Following that, a risk model for predicting IPF prognosis was developed utilizing multiple stepwise Cox regression analyses of 3 glycolysis-related genes, which was then validated using the GSE27957 and GSE70866 cohorts. The ROC curve analysis illustrated that the 3 glycolysis-related genes signature was fitted for forecasting dismal prognosis in IPF patients. Furthermore, the 1, 2, and 3-year OS rates were intuitively predicted using a nomogram. This predictive model centered on these 3 glycolysis-related genes is both monetarily cost-effective and clinically advantageous. Lu et al^[44]^ also developed a nomogram using the 3 inflammation-associated genes in IPF. We employ several bioinformatics analytic approaches that are similar to some of the procedures used by Lu et al.

This prediction method, however, was discovered to have some limitations. Firstly, the model was built using the GEO dataset; thus, it needed to be validated in a clinical patient population. Also, the number of patients included in our study was small. Further expansion of the sample size is required to validate this model in the future. Secondly, because this was retrospective research, more prospective studies are required to confirm the findings. Lastly, functional studies are required to additionally uncover the possible mechanism of glycolysis-related genes that might be important in IPF.

## 5. Conclusion

Ultimately, following a thorough bioinformatics analysis of the GEO database, our investigation discovered 3 IPF prognostic glycolysis-related genes. The findings provided new insights into the functions performed by glycolysis in IPF pathogenesis, perhaps paving the way for the creation of prognostic indicators.

## Author contributions

**Conceptualization:** Yu Li, Yaju Deng, Jie He.

**Data curation:** Yu Li, Jie He.

**Formal analysis:** Yaju Deng, Jie He.

**Investigation:** Jie He.

**Methodology:** Jie He.

**Project administration:** Yaju Deng, Jie He.

**Resources:** Yu Li, Yaju Deng, Jie He.

**Software:** Yu Li, Jie He.

**Supervision:** Jie He.

**Validation:** Yu Li, Jie He.

**Writing – original draft:** Yu Li, Yaju Deng.

**Writing – review & editing:** Yu Li, Yaju Deng.

## Supplementary Material
