# Supplementary material for: A novel prognostic index based on the analysis of glycolysis-related genes in idiopathic pulmonary fibrosis
Source: Medicine (Baltimore). 2023 Mar 17;102(11):e33330. doi: 10.1097/MD.0000000000033330 (PMC10019186; doi:10.1097/MD.0000000000033330)
Supplement: Supplementary file 1 [file medi-102-e33330-s001.pdf]

Supplementary Table 1. 255 glycolysis-related genes

Gene
EFNA3
ERO1A
SPAG4
KIF20A
DEPDC1
HMMR
GMPPA
B3GNT3
CENPA
KDELR3
PMM2
SLC25A10
AURKA
COG2
NUP155
GFPT1
GAPDH
LDHA
B4GALT2
ABCB6
NUP85
CHPF2
GPC3
CDK1
SLC25A13
CASP6
HSPA5
PGM2L1
GPI
TSTA3
TPI1
RPE
PSMC4
ENO1
SLC37A4
PC
COPB2
TPBG
ALDOA
HS6ST2
PFKP
RAE1

NDC1
FUT8
PKM
HK3
B4GALT4
PPP2CB
SEC13
B4GALT7
MIOX
MDH2
CHPF
PAXIP1
FKBP4
NUP43
PLOD1
SRD5A3
XYLT2
GOT1
DCN
NUP88
BIK
B3GAT3
CD44
GALE
NUP62
SDC2
COL5A1
EGLN3
HDLBP
BPNT1
IGFBP3
POLR3K
MIF
NUP35
ARTN
NUP107
CYB5A
ALG1
MPI
ANKZF1
GOT2
HAX1
NUP42
NUP37

CITED2
AK4
P4HA1
SDHC
NUP210
NASP
PPFIA4
PPIA
SLC16A3
CAPN5
NOL3
AAAS
PLOD2
CTH
NUP205
CLDN3
KIF2A
TKTL1
IDH1
PGK1
GMPPB
PGP
VCAN
NUP93
ECD
TGFA
NUP133
PFKFB2
TXN
FAM162A
SOD1
VLDLR
GCKR
NANP
AKR1A1
GCLC
TALDO1
GNPDA1
PGAM1
GPR87
TPR
SDC1
SEH1L
PFKL

NSDHL
PPP2R5D
ELF3
STMN1
AK3
PYGB
IER3
ARPP19
NT5E
RRAGD
NUP50
ENO3
PRKACA
GYS1
PPP2R1B
UGP2
DDIT4
ADORA2B
B4GALT1
GLRX
ANGPTL4
PPP2R1A
ANG
LCT
GALK1
DPYSL4
LHX9
GUSB
HOMER1
PRPS1
ENO2
AGRN
LHPP
GCK
NUP160
NUP54
B3GALT6
PFKFB4
NDUFV3
DSC2
MDH1
SAP30
PFKFB3
PGLS

NUP188
FBP2
CHST6
POM121C
CLDN9
G6PD
PKP2
CACNA1H
ME2
ALDOC
TPST1
PGK2
IDUA
MET
HS2ST1
PYGL
PKLR
IRS2
ISG20
ALDH7A1
PAM
RBCK1
NUP58
GAPDHS
CLN6
POM121
HK2
ZNF292
SOX9
PDK3
MED24
SLC35A3
CHST4
NUP153
PFKFB1
ALDH9A1
GAL3ST1
PRKACG
NUP98
B3GAT1
MXI1
GPC1
GNE
PHKA2

DLD
PRKACB
QSOX1
VEGFA
AGL
TFF3
BPGM
EXT2
GLCE
ME1
PGM2
PPP2CA
TGFBI
IL13RA1
STC2
GNPDA2
HK1
MERTK
SDC3
CHST12
EXT1
NUP214
LDHC
NDST3
ADPGK
GALK2
CXCR4
P4HA2
GPC4
ALDOB
GYS2
STC1
EGFR
PFKM
CHST2
RANBP2
CHST1
