# Supplementary material for: A novel prognostic index based on the analysis of glycolysis-related genes in idiopathic pulmonary fibrosis
Source: Medicine (Baltimore). 2023 Mar 17;102(11):e33330. doi: 10.1097/MD.0000000000033330 (PMC10019186; doi:10.1097/MD.0000000000033330)

Supplementary Figure 1: we conducted a risk score analysis of the three glycolysis-associated genes that were included in the prognostic model in the GSE70866 cohort: (A) PCA plot for IPF patients premised on the risk score in the GSE70866 cohort, (B) Survival plots for patients classified into high- or low-risk groups in the GSE70866.cohort, (C) Prediction of the overall survival in the GSE70866 cohort using the ROC curve.

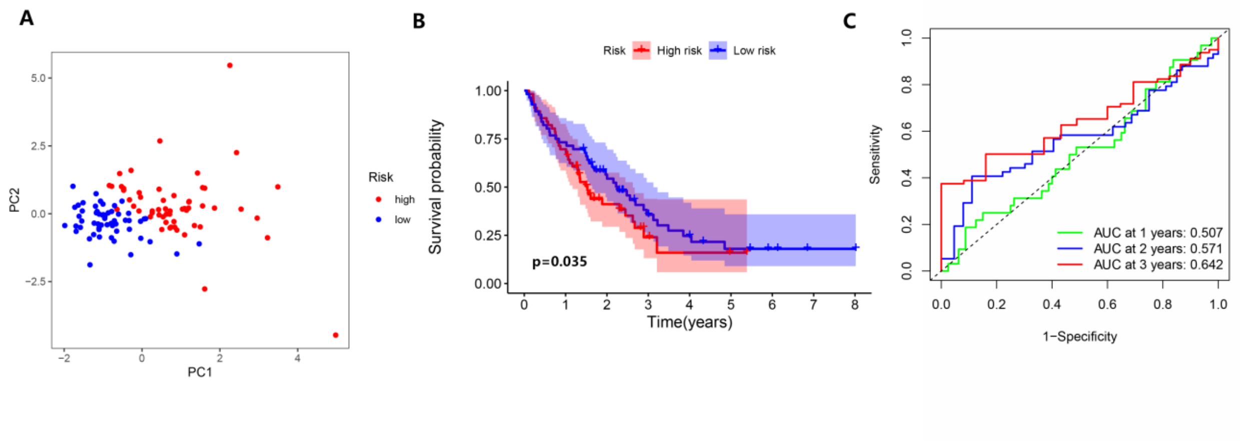

Supplement: Supplementary file 2 [file medi-102-e33330-s002.pdf]
